# Supplementary material for: A Critical View of the Application of the APEX Software (Aqueous Photochemistry of Environmentally-Occurring Xenobiotics) to Predict Photoreaction Kinetics in Surface Freshwaters
Source: Molecules. 2019 Dec 18;25(1):9. doi: 10.3390/molecules25010009 (PMC7017383; doi:10.3390/molecules25010009)
Supplement: Supplementary file 1 [file molecules-25-00009-s001.zip › 3.2.4_gcc-4.4.0.zip!/3.2.4_gcc-4.4.0/3.2.4_gcc-4.4.0/doc/octave/html/interpreter/Function-Overloading.html]

Function Overloading - Untitled


Next: Operator Overloading,
Up: Overloading Objects


---

#### 33.4.1 Function Overloading

Any Octave function can be overloaded, and allows a object specific
version of this function to be called as needed. A pertinent example
for our polynomial class might be to overload the `polyval` function
like

```
```
     function [y, dy] = polyval (p, varargin)
       if (nargout == 2)
         [y, dy] = polyval (fliplr(p.poly), varargin{:});
       else
         y = polyval (fliplr(p.poly), varargin{:});
       endif
     endfunction
```
```

This function just hands off the work to the normal Octave `polyval`
function. Another interesting example for an overloaded function for our
polynomial class is the `plot` function.

```
```
     function h = plot(p, varargin)
       n = 128;
       rmax = max (abs (roots (p.poly)));
       x = [0 : (n - 1)] / (n - 1) * 2.2 * rmax - 1.1 * rmax;
       if (nargout > 0)
         h = plot(x, p(x), varargin{:});
       else
         plot(x, p(x), varargin{:});
       endif
     endfunction
```
```

which allows polynomials to be plotted in the domain near the region
of the roots of the polynomial.

Functions that are of particular interest to be overloaded are the class
conversion functions such as `double`. Overloading these functions
allows the `cast` function to work with the user class and can aid
in the use of methods of other classes with the user class. An example
`double` function for our polynomial class might look like.

```
```
     function b = double (a)
       b = a.poly;
     endfunction
```
```
